# The Application of Inorganic Nanoparticles in Molecular Targeted Cancer Therapy: EGFR Targeting

**DOI:** 10.3389/fphar.2021.702445

**Published:** 2021-07-12

**Authors:** Meng Sun, Ting Wang, Leijiao Li, Xiangyang Li, Yutong Zhai, Jiantao Zhang, Wenliang Li

**Affiliations:** ^1^ School of Chemistry and Environmental Engineering, Changchun University of Science and Technology, Changchun, China; ^2^ Department of Colorectal and Anal Surgery, The First Hospital of Jilin University, Changchun, China; ^3^ Jilin Collaborative Innovation Center for Antibody Engineering, Jilin Medical University, Jilin, China

**Keywords:** inorganic nanoparticles (iNPs), epidermal growth factor receptor (EGFR), molecular targeted, multifunctional nanotherapeutics, cancer treatment

## Abstract

Epidermal growth factor receptor (EGFR) is an anticancer drug target for a number of cancers, such as non-small cell lung cancer. However, unsatisfying treatment effects, terrible side-effects, and development of drug resistance are current insurmountable challenges of EGFR targeting treatments for cancers. With the advancement of nanotechnology, an increasing number of inorganic nanomaterials are applied in EGFR-mediated therapy to improve those limitations and further potentiate the efficacy of molecular targeted cancer therapy. Given their facile preparation, easy modification, and biosecurity, inorganic nanoparticles (iNPs) have been extensively explored in cancer treatments to date. This review presents an overview of the application of some typical metal nanoparticles and nonmetallic nanoparticles in EGFR-targeted therapy, then discusses and summarizes the relevant advantages. Moreover, we also highlight future perspectives regarding their remaining issues. We hope these discussions inspire future research on EGFR-targeted iNPs.

## Introduction

Cancer is a major public health problem worldwide (Li et al., 2021a). The incidence and mortality rates are on the rise year by year all over the world (Bray et al., 2018; Li et al., 2021b; Siegel et al., 2021). As we know, the conventional treatment methods for malignant tumors are surgery, chemotherapy, and radiotherapy. However, the finite antineoplastic effects also associate with some serious side effects for patients with cancer due to the destructiveness generated by the conventional treatment strategies, such as pain, partial loss of bodily function, and complications (Pearce et al., 2017; McGhee and Steele, 2020). Molecular targeted therapy is an emerging cancer therapy strategy that possesses specific anticancer effects at cellular and molecular levels (Lee et al., 2018; Goyal et al., 2021). It could be used to identify specific cancerogenic targets of tumor cells or microenvironment (TME) and thereby provide negative control of the signaling pathways associated with cell proliferation and metastasis (Yang et al., 2021). On the one hand, the significant inhibition of tumor cells’ growth and metastasis is accomplished. On the other hand, the immune response could be simultaneously activated by molecular targeted drugs (Liu et al., 2020). Therefore, molecular targeted therapy has become the standard cancer treatment strategy for many malignant tumors due to it being more effective for tumor cells and having fewer side effects for normal cells (La Salvia et al., 2021).

Tumor tissue is mainly composed of parenchyma and stroma. Tumor parenchyma essentially refers to carcinoma cells, which has specificity in relation to tissue. Carcinoma cells are characterized by intense proliferation due to the insensitiveness to apoptotic signal, escaping from apoptosis and so on. The tumor stroma, namely TME, provides survival necessity and conditions for tumor cell growth and metastasis (Wu and Dai, 2017). TME consists mainly of stromal cells, extracellular matrix, and other extracellular regulatory factors. Cell tumor-associated fibroblasts, immune cells, and vascular endothelial cells are all belong to stromal cells, which provide multifarious specific targets for molecular targeted agents. Due to the said factors, the conventional molecular targeted agents aim at targeting tumor cells and TME prevailingly.

Epidermal growth factor receptor (EGFR) is a transmembrane protein that widely distributes in epithelial cells, fibroblasts, spongiocytes, and keratinocytes. EGFR plays a crucial role in cell growth metastasis and angiogenesis (Mendelsohn and Baselga, 2000). Frequent abnormalities in the expression of EGFR and EGFR-mediated activation of downstream signaling pathways have been detected in many human malignancies. Anti-EGFR targeted therapy has been brought into focus in recent years (Chen et al., 2020a; Fasano et al., 2021). To date, two primary species of EGFR targeted agents include monoclonal antibodies (McAb) and small molecule tyrosine kinase inhibitors (TKI). McAb act on the extracellular region of the receptor, while TKI act on the intracellular region of the receptor. The most commonly used McAb is cetuximab (C225) (Van Cutsem et al., 2009). Moreover, several EGFR-TKI have been approved by the FDA to date including gefitinib, erlotinib, lcotinib, afatinib, lapatinib, osimertinib, and vandetanib (Westover et al., 2018). Unfortunately, the present preclinical and clinical data show a low cure rate, easy recurrence, and adverse events (Paez et al., 2004). For example, EGFR inhibitor brings various awful gastrointestinal, hematologic, and endocrine disorders, arthritis, mucositis, and rash, restricting their wide application (Vermorken et al., 2007; Bar-Ad et al., 2016). Drug resistance is another common problem in the treatment of EGFR-targeted drugs, such as the changes of miRNA and gene profiles (Holohan et al., 2013). As reported, a second point mutation happens in the DNA sequence of the EGFR gene in patients with non–small-cell lung cancer at relapse due to the obvious gefitinib resistance (Kobayashi et al., 2005). In addition, resistant subclones containing an additional EGFR mutation has been observed in cancer patients bearing erlotinib-sensitive EGFR mutations (Pao et al., 2005). More importantly, poor water solubility and insufficient accumulation of EGFR inhibitors at the tumor site limit their application. Therefore, all kinds of new methods and ways are explored actively so as to solve the above problems.

**FIGURE 1:**
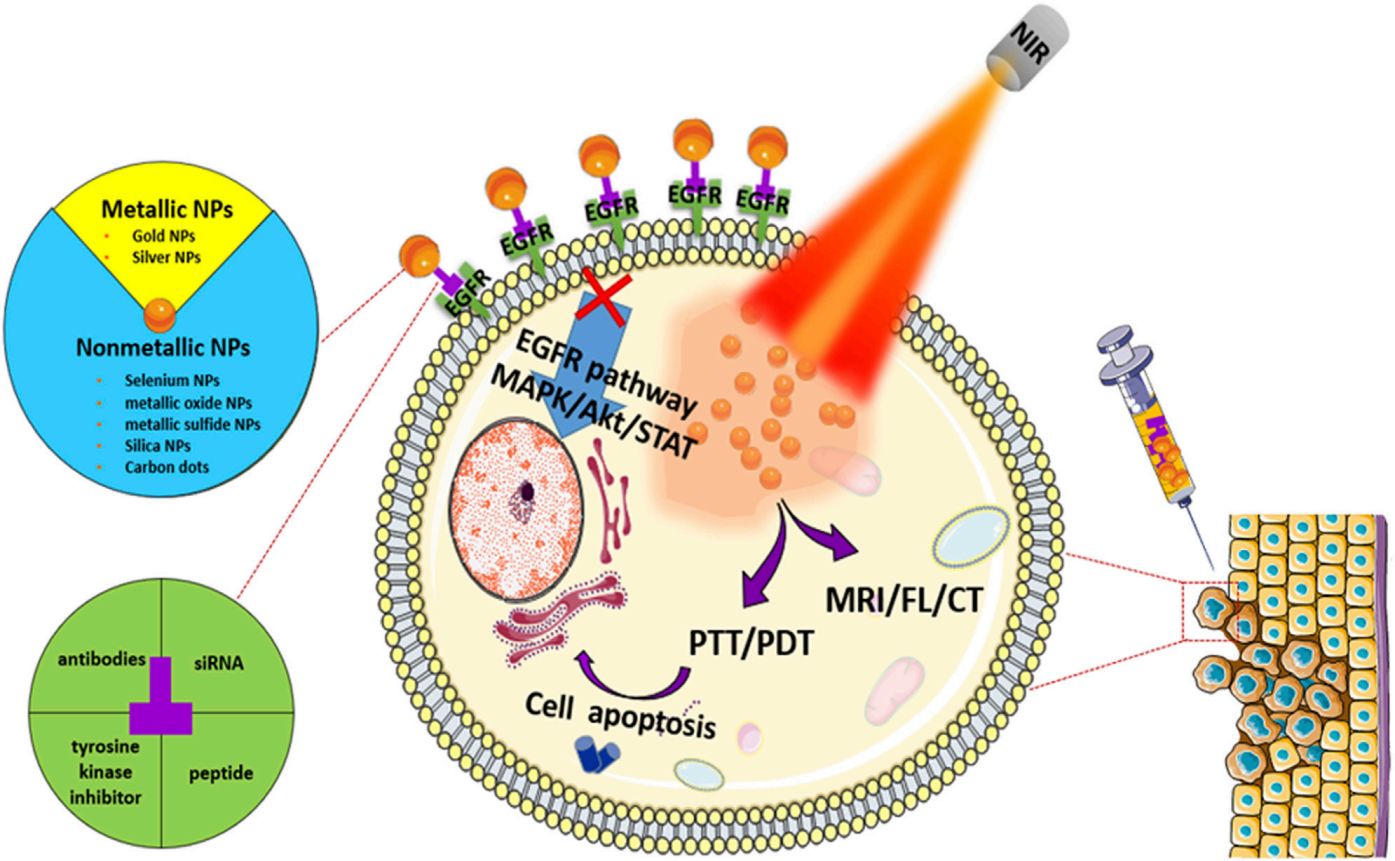
Schematic illustration of the main topics covered in this review.

The use of modern nanoscience and material science has provided new approaches to conquer cancer (Chen et al., 2020b; Er Saw and Jiang, 2020; Zhao et al., 2020). Novel nanoparticle engineering is considered as a major innovative impetus in combining diagnosis and treatment into an integrated nanoplatform known as “nanotherapeutics.” INPs, important carriers, can lower the drug dose, prolong retention time, and achieve targeted delivery, thus increasing the cure rate and reducing complications. Moreover, iNPs could change the immunosuppressive environment. Therefore, iNPs could effectively deliver and extensively accumulate EGFR-targeted drugs in tumor tissue, reducing the accumulation of drugs in normal tissues. The interaction between EGFR and karyopherin-β does not interfere with the EFGR-bonded nanoparticles. Combining inorganic nanoparticles with EGFR-targeting can improve the effectiveness and compatibility, change drug resistance of EGFR-targeting drugs, and combine multiple therapeutic approaches on a single nanoplatform to achieve synergistic therapeutic effects. In this review, some bioactive iNPs, such as the McAb and EGFR-KTI drug deliveries, are focused on and summarized with emphasis on their applications in tumor therapy (Table 1 and Figure 1), in order to provide new ideas for subsequent research.

**TABLE 1:** Summary of the Inorganic nanoparticle for EGFR-targeted therapeutic strategies.

Nanoparticles	Targeted therapy strategies	Strengths	References
Au NPs	1) Au NPs as vehicles for EGFR antibodies and KTIS.	AuNPs are easily functionalized, stable, low toxic, biocompatible, possess a large surface area for drug attachment, and enable fluorescence and photoacoustic chromatography imaging, controlled drug release, and photothermal therapy. EGFR inhibitors and tumor-targeting peptides can target cancer cells and block signaling pathway	25–31
Ag NPs	2) AuNPs conjugate with tumor targeting peptides	AgNPs has the effect of radiosensitization. EGFR-specific small molecules or EGFR mAb can target tumor cells and inhibit EGFR signal	32–33
Se NPs	Incorporation of the EGFR-specific small molecules or EGFR mAb into Ag NPs	SeNPs has the functions of anticancer, immunomodulatory and drug carrier. EGFR-targeted elements make nanoparticles EGFR targeted, increase SeNPs uptake and inhibit tumor cell growth/survival, metastasis and angiogenesis	34–36
IONPs	Combination of Se NPs and various EGFR-targeted elements (SiRNA, peptides, antibody)	IONPs has unique optical and magnetic properties and can be used in drug delivery, laser hyperthermia, MRI, radiotherapy and PDT.	39–44
MxS or MxSe (x = 1–2)	1) EGFR-conjugated IONPs	EGFR antibody provides specific targeting. Composite platform combines multiple treatment modalities. Novel carbon materials have a considerable specific surface area can be used as drug carriers	45–50
Metallic oxideare	2) EGFR-targeted composite nanoplatforms	ZnS QDs, AgS QDs, CdSe QDs can be used as fluorescent probes, and CuS NPs can be used for photothermal treatment. EGFR antibody provides specific targeting. Their combination can achieve EGFR targeted imaging or photothermal synergistic therapy	56
SNs	3) Novel carbon materials participate in the construction of multifunctional therapeutic agents	ZnO is pH sensitive and allows for controlled release of Zn2+ and loaded drugs in the tumor microenvironment. EGFR antibody or EGFR KTI provides specific targeting and inhibition. Nanotheranostic platforms can realize synergistic therapy	59–73
CQDs	1) Metal sulfide combined with EGFR antibody (ZnS, CuS, AgS)	SNs can be used as nanocarriers due to their excellent biodegradability, high porosity and surface area. EGFR-targeted SNs further realize targeted delivery. Specific drug can be used for chemotherapy. siRNA can silence tumor-related genes and reverse multidrug resistance of cancer. Nano-contrast agents can be used for real-time tumor detection	80–84
	2) Metal selenide combined with EGFR antibody (CdSe)	CQDs has photostability, chemical stability and low toxicity, so it can be used for biological imaging. EGFR antibody modification provides specific targeting. The addition of CT or MRI reagents can improve the spatial resolution of fluorescence imaging and enhance tissue penetration	
	Metallic oxideare establish nanotheranostic platforms combining with EGFR antibody or EGFR KTI (ZnO)		
	1) EGFR-targeted SNs delivery specific drug		
	2) EGFR-targeted MSNs deliver siRNA.		
	3) EGFR-labeled MSNs used as directional carriers of nano-contrast agents		
	Construction of multifunctional nano-platform based on EGFR-targeted CQDs combined contrast agent, SFN, MIP.		

## Metallic Nanoparticles

Recent years have witnessed the wide application of oncotherapy of iNPs. Due to their particular physico-chemical properties, metallic NPs (MNPs) have been explored in EGFR-targeted therapy to achieve synergistic treatment, improve therapeutic effects, and delay the development of drug resistance. These multifarious adopted strategies in EGFR-targeted therapy are summarized as follows.

### Gold Nanoparticles

**FIGURE 2:**
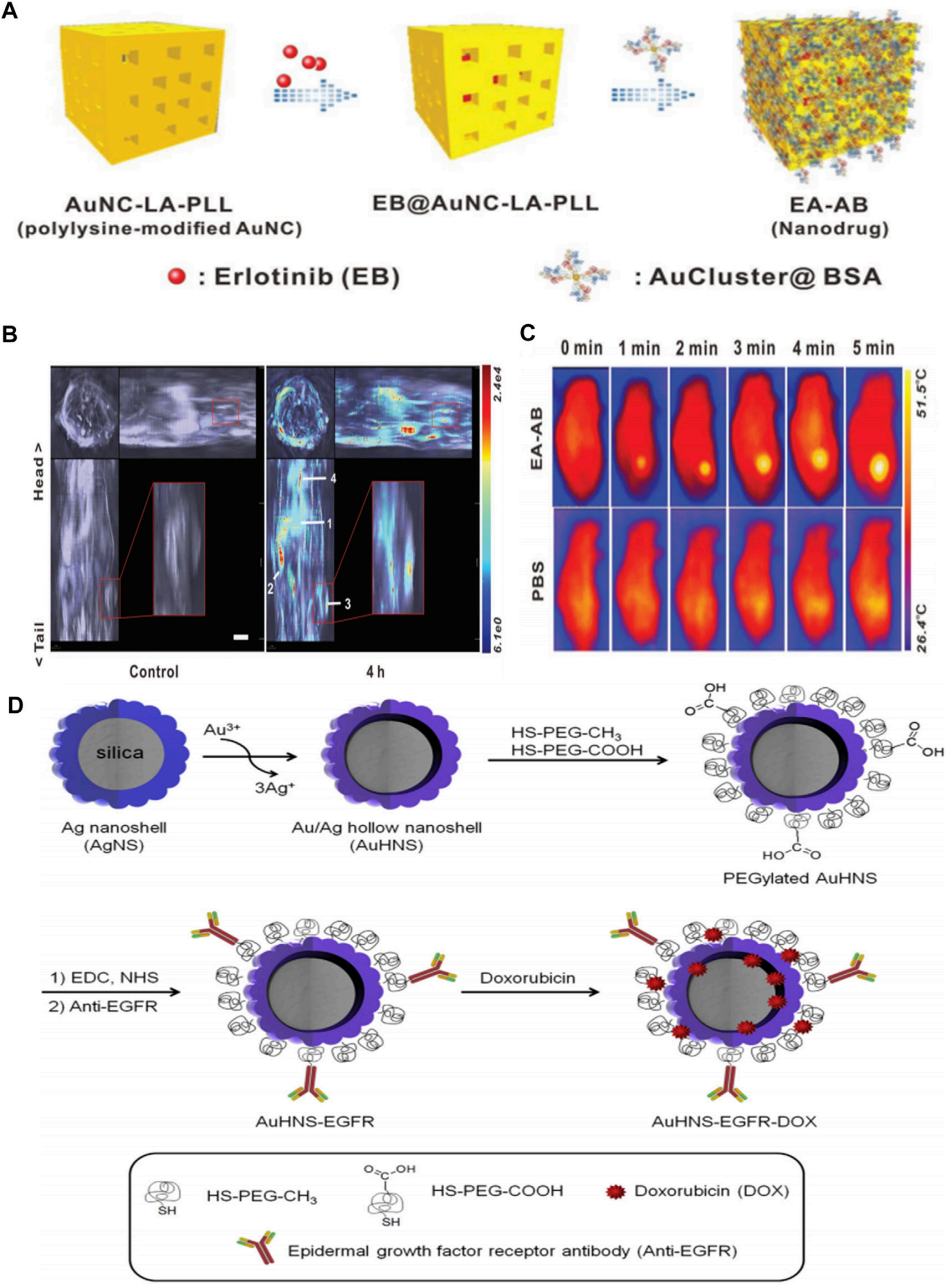
**(A)** Schematic illustration for fabrication of EA–AB **(B)** *In vivo* orthogonal 3D MSOT view of 4T1 tumor-bearing mice before and after tail vein injection of the EA–AB dispersion (AuNC content: 80 mg kg^−1^). The numbers indicate different organs and tumor regions: 1) liver, 2) spleen, 3) tumor, and 4) lung **(C)** Infrared thermal images of 4T1 tumor-bearing mice intravenously injected with 200 µL PBS (the control) or EA–AB dispersion (AuNC content: 80 mg kg^−1^) and 12 h later subject to 5 min of NIR laser irradiation (808 nm at 1.5 W cm^−2^). Reproduced from (Avvakumova et al., 2019) with permission from Wiley **(D)** Schematic illustration for the preparation of anti-EGFR-conjugated and doxorubicin-loaded Au/Ag hollow nanoshell (AuHNS-EGFR-DOX). Reproduced from (Yu et al., 2017) with permission from Elsevier.

Gold NPs (Au NPs) are one of the most studied diagnosis and treatment agents for the treatment of cancer due to their ease of functionalization, good stability, low toxicity, and high biocompatibility. Au NPs-based targeted nanomedicine, such as AurImmune™ (CYT-6091, Au-rhTNF), has entered clinical trials. Au NPs have been demonstrated as EGFR antibodies and KTIs’ vehicles in molecule targeted therapy because of their large surface area for attachment of targeting drugs. For example, EGF-tagged Au NPs, as a versatile delivery, was bound to indium-111 to form an ^111^In-labelled EGF-targeted agent (^111^In-EGF-Au-PEG) (Song et al., 2017). The outcomes showed that ^111^In-EGF-Au-PEG could effectively and specifically target EFGR-positive cancer cells (MDA-MB-468), enhance the tumor uptake, and reduce liver uptake compared to the unlabeled ^111^In- Au-PEG NPs. Au NPs are also qualified for the delivery of EGFR antibodies and cetuximab is the most widely used and reliable EGFR antibody. Groysbeck et al. conjugated water-soluble thiolate-protected Au NPs with cetuximab (AuNP-Cetuximab) against EGFR expressing glioblastoma cells. The obvious inhibition of EGFR autophosphorylation was observed (Groysbeck et al., 2019). The conjugated Au NPs endowed their electronic properties without any influence on the biological behavior of cetuximab. Additionally, the antineoplastic activity of cetuximab-conjugated cubic gold nanocages (Au NCs) for EGFR targeting in triple-negative breast cancer cells have been studied by Prosperi’s group (Avvakumova et al., 2019). The improved permeability and retention effect of AuNCs resulted in active targeting. They concluded that the conjugation strategy of binding AuNPs to EGFR antibody was an important factor that affected the effectiveness of cellular uptake and the active feature of AuNPs in cancer cells. Wu et al. used bovine serum albumin (BSA) stabilized Au nanocluster (AuCluster@BSA for short) as an EGFR inhibitor delivery. This strategy was not restricted to transfer erlotinib directionally, but could realize whole-body multispectral optoacoustic tomography imaging and photothermal therapy with the aid of Au cluster (Zhan et al., 2019) (Figures 2A–C).

Moreover, recent research showed that AuNPs could conjugate with some peptide resulting in fascinating EGFR-targeting functionality, such as artificial peptide GE11 decorate AuNPs. Yu et al. conjugated Au NPs with different functional polypeptides for tumor-targeting gene therapy (Yan et al., 2020). These composite nanosystems (AuNPPs) consisted of Au NPs, targeting peptide GE11, cell-penetrating peptide octaarginine (R8), and polyhistidine. The resultant AuNPPs were endowed with tumor targeting and redox-responsive features. The vitro and vivo experiment results showed that AuNPPs possess great tumor cell-targeting ability, transfection efficiency, and tumor growth suppression. Au cluster could further be modified by both the specific GE11 peptide (YHWYGYTPQNVI) and peptide (Au_10_Peptide_5_-GE11: CCYKKKYHWYGYTPQNVI) to achieve cancer inhibition through dual pathways. On one hand, the designed Au_10_Peptide_5_-GE11 could target EGFRs in both active and inactive states, and then induce the oxidative stress mediated apoptosis in the active EGFR mediated endocytosis process. On the other hand, the inactive state of EGFR at the membrane of tumor cells could be maintained by binding Au_10_Peptide_5_-GE11 to avoid the dimerization and further inhibit the activities of tumor cells. Therefore, employing Au_10_Peptide_5_ as EGFR TKIs delivery results in inhibiting cancer cells through dual pathways (Zhang et al., 2018a).

### Silver Nanoparticles

In addition to Au NPs, silver nanoparticles (Ag NPs) have been recognized as useful tools for diagnosis and treatment in anticancer and antimicrobial fields. Ag NPs were found to have potential utility as radiosensitizers to improve the outcomes of cancer radiotherapy. Therefore, the incorporation of the EGFR-specific small molecules (e.g. gefitinib) or humanized McAb targeting EGFR (e.g. Erbitux; C225) into Ag NPs is an alternative strategy. For instance, C225-coated Ag NPs (Ag/C225) have been tested for their enhanced radiosensitization in nasopharyngeal carcinoma epithelial cell lines (Yu et al., 2017). The anti-EGFR antibody activity was well maintained in Ag/C225 nanocomposite with the average preserved activity of about 82%. More importantly, Ag/C225 nanocomposites were not cytotoxic alone for normal cell lines without X-ray irradiation. According to the half maximal inhibitory concentration values (IC_50_), Ag/C225 nanocomposites cause irreversible cell growth inhibition. Therefore, Ag/C225 nanocomposites, as a specific radiosensitizer, exhibited better anti-proliferative effects in nasopharyngeal carcinoma cell lines by assistive EGFR-targeting of C225. Cho et al. developed a targeted Au/Ag drug delivery (AuHNS-EGFR-DOX) for near-infrared (NIR) light induced thermo-chemotherapy (Noh et al., 2015). The Au/Ag hollow nanoshells were prepared on PEGylation silica NPs (AuHNS) to further conjugate EGFR antibody. There is a hollow interior between the Au/Ag shell and the silica core particle with the distance of about 16 nm to load doxorubicin (DOX). The DOX loading capacity was about 3×10^6^ DOX molecules per single nanoparticle. The targeted-drug delivery was relatively stable at pH7.4, while the DOX release rate was 12% over 24 h at pH5.0. Moreover, DOX release could be triggered by the NIR-induced hyperthermia. This EGFR-targeted thermo-chemotherapic agent was approved to be a pH-sensitive and thermo-sensitive drug release system. Additionally, the A549 cell viability was much lower in AuHNS-EGFR-DOX group under NIR irradiation than that in AuHNS- DOX group, indicating the marked availability of EGFR-mediated endocytosis compared to non-specific cellular uptake. These works indicated that metallic nanoparticles, such as McAb or EGFR-KTI deliveries, extended the application of metallic nanoparticles in targeting therapy for tumor (Figure 2D).

## Non-metallic Nanoparticles

In this section, we selectively present various typical nanometallic nanoparticles that have been synthesized and conjugated to EGFR-targeted small molecule drugs for numerous oncotherapies in recent years. The applications in oncotherapy of non-metallic nanomaterials consisting mainly of selenium (Se), iron-based nanomaterials, metallic chalcogenide, metallic oxideare, silicon, and carbon-based materials have been described in the following sections.

### Selenium Nanoparticles

**FIGURE 3:**
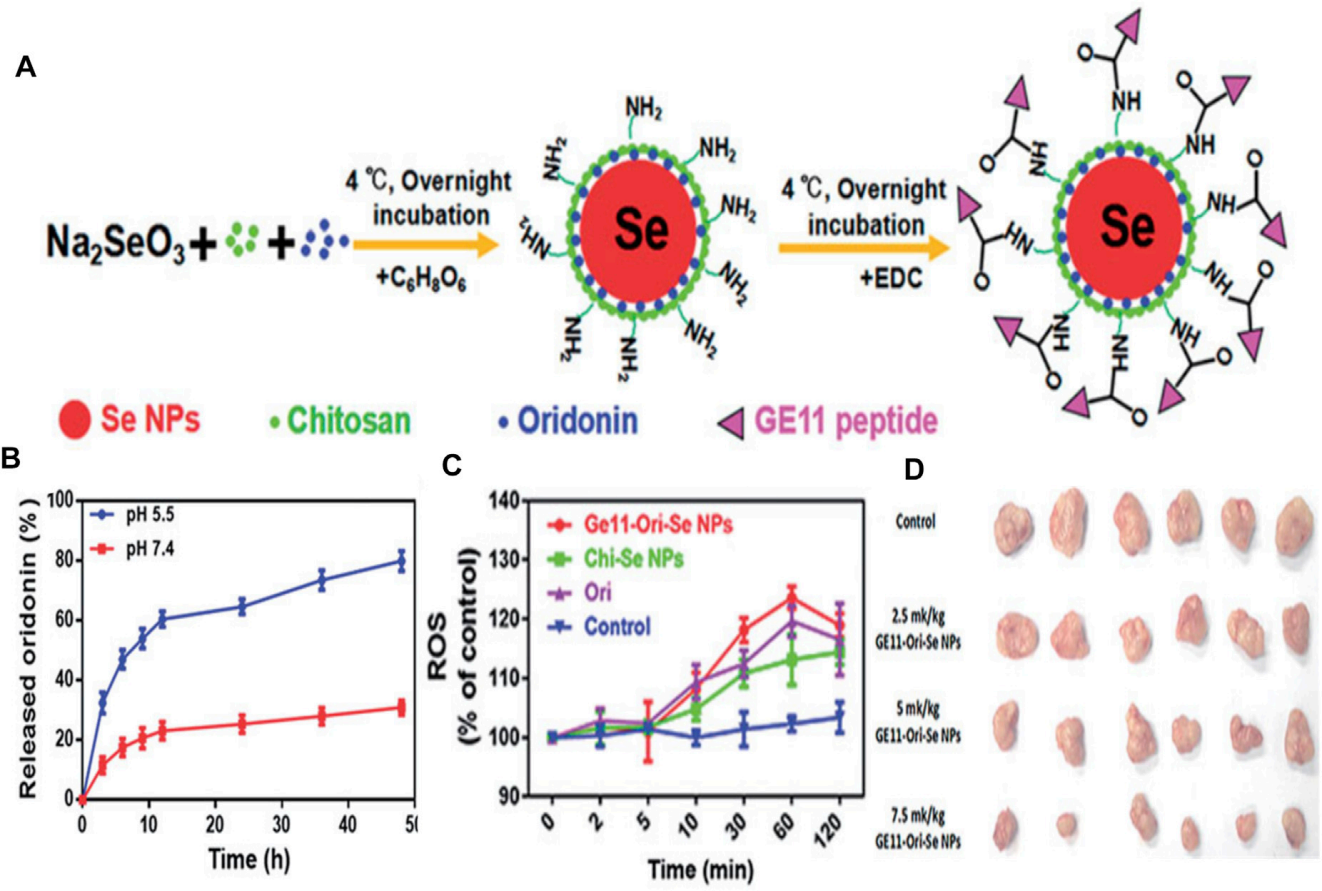
**(A)** The formation process of GE11-Ori-Se NPs **(B)** *In vitro* release of oridonin from GE11-Ori-Se NPs at pH 5.5 and 7.4 **(C)** Effects of GE11-Ori-Se NPs, and the same dosage of oridonin or Chi-Se NPs on the production of ROS in KYSE-150 cells **(D)** Images of the tumor from control and GE11-Ori-Se NPs treated xenograft KYSE-150 cancer nude mice. Reproduced from (Kong et al., 2011) with permission from Informa.

Se elements with anticancer and immunoregulation properties are indispensable and necessary in humans. Gao’s team found that selenium nanoparticles could inhibit the growth of prostate cancer cells in part through cystepsin mediated apoptosis and Ahmadrezashahverdi’s team demonstrated that Se nanoparticles could significantly induce the immune response of 4T1 breast cancer tumors in mice (Kong et al., 2011; Yazdi et al., 2012). Consequently, the combination of Se NPs and various EGFR-targeted elements have been used recently. For instance, Jebal et al. synthesized hexagonal selenium nanoparticles and then endowed them with EGFR-targeting abilities by modifying SiRNA (HSNM-SiRNA) (Moghaddam et al., 2016). HSNM-SiRNA was found to be able to change the structure of EGFR and destroy its activity when NSCLC cells were exposed to HSNM-SiRNA. Besides, GE11 peptide-conjugated Se NPs (GE11-Ori-Se NPs) were applied as oridonin delivery to inhibit growth and metastasis of EGFR over-expressed cancer cells and reduce the toxicity against normal cells (Pi et al., 2017). Substantial oridonin molecules could release in a tumor acidic microenvironment and thus enter into the lysosomes and cytoplasm of cancer cells. Impressively, GE11-Ori-Se NPs could trigger reactive oxygen species (ROS) production. And GE11-Ori-Se NPs further killed cancer cells by disrupting the function of mitochondria, specifically inhibiting EGFR-mediated PI3K/AKT and Ras/Raf/MEK/ERK pathways (Figures 3A–D). Chen and co-workers recently performed anti-EGFR therapy against nasopharyngeal carcinoma by combining Se NPs with gefitinib, which is a human-mouse chimeric antibody blocking EGFR. This strategy effectively increased intracellular accumulation in nasopharyngeal carcinoma cells with the help of selenium nano-platform and thus sheds light on its application in anti-EGFR therapy (Huang et al., 2019).

### Iron Oxide Nanoparticles

Iron is another indispensable microelement in the human body. Iron is required in multitudinous biological processes such as composition of hemoglobin to deliver oxygen around the body. Importantly, iron-based nanomaterials have been approved by the FDA in inorganic nanomedicines (Bobo et al., 2016). Iron oxide nanoparticles (IONPs) are well established nano-therapeutic platforms in clinical trials against several types of cancer. Because of the peculiar optical and magnetic properties, IONPs are widely used in laser-induced thermotherapy, magnetic resonance imaging (MRI), radiotherapy, and photodynamic therapy (PDT) (Zhang et al., 2016).

**FIGURE 4:**
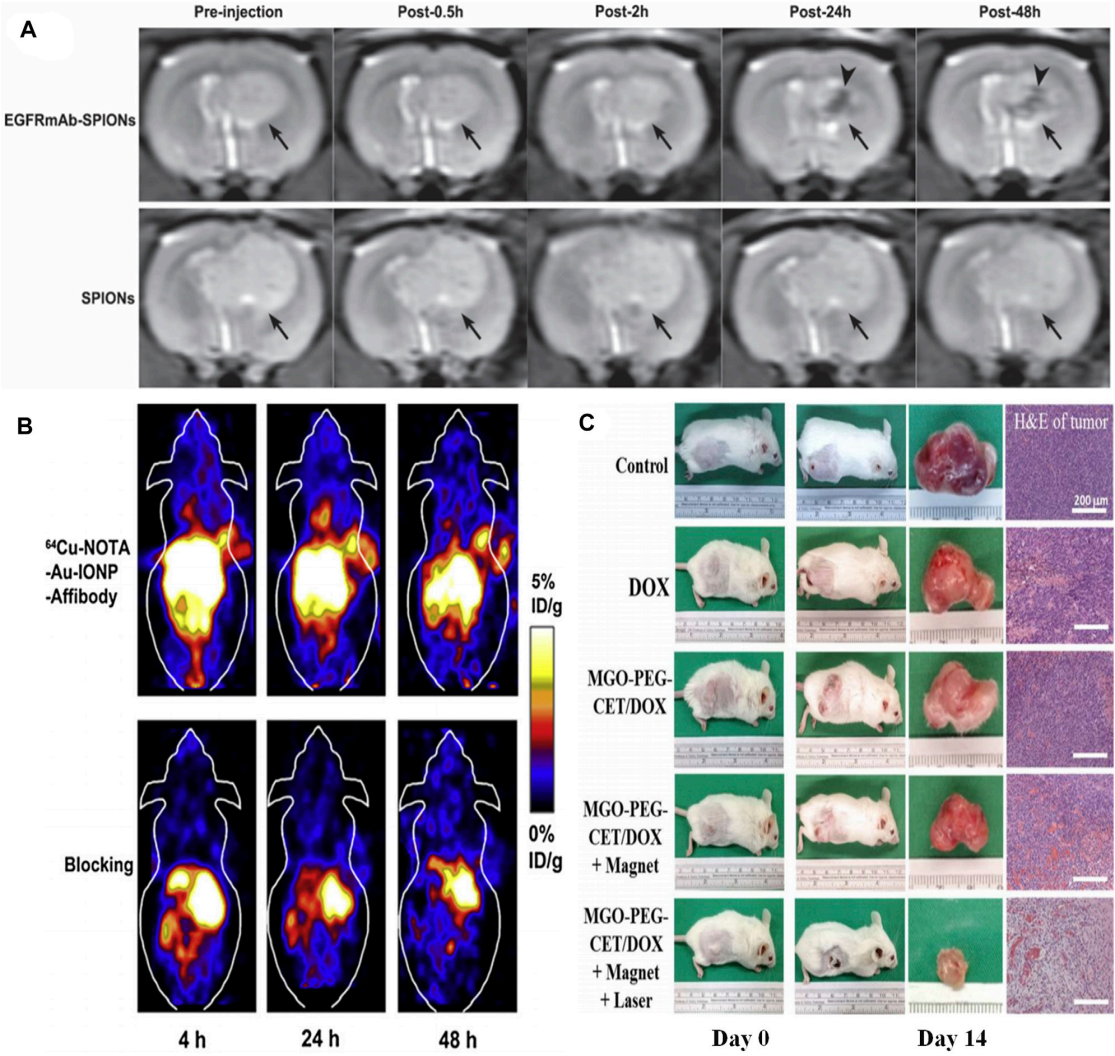
**(A)** *In vivo* T2-weighted images of rat brain bearing C6 glioma (arrow) after administration of EGFRmAb-SPIONs **(upper row)** and SPIONs **(lower row)**. Reproduced from (Huang et al., 2019) with permission from SAGE Publications **(B)** *In vivo* decay-corrected whole body coronal PET images of A431 tumor bearing mice acquired 4, 24 and 48 h after injection of ^64^Cu-NOTA-Au-IONP-Affibody and the blocking dose of Affibody. Reproduced from (Bobo et al., 2016) with permission from Elsevier **(C)** The gross observation of tumor-bearing BALB/C mice on day 0 and 14, the gross view of incised tumor and the H&E staining of the incised tumor on day 14 (bar = 200 µm). Reproduced from (Yang et al., 2013) with permission from Multidisciplinary Digital Publishing Institute.

As for MRI agents, EGFR-conjugated superparamagnetic Fe_3_O_4_ (EGFRmAb-SPIONs) were synthesized and served as a targeted MRI contrast agent for the EGFR-positive detection *in vitro* and *in vivo*. The MRI results suggested that the brain glioma cells treated with EGFRmAb-SPIONs exhibited a more significant negative contrast enhancement even at low concentrations than that observed in the tumor cells incubated only with SPIONs (Mu et al., 2015). The dramatic reduction of T_2_ relaxation time was found as a result of the treatment of EGFRmAb-SPIONs. According to the *in vivo* experimental results, T_2_-weighted MRI exhibited an obvious hypointense region within glioma after intravenous administration of EGFRmAb-SPIONs. The maximal negative enhancement within the tumor was reached at 24 h after injection along with the increasing R_2_ value (Figure 4A).

A number of EGFR-targeted composite nanoplatforms with IONPs as the main component for targeting MRI and therapy have been studied so far. Cheng’s group developed a hetero-nanostructural trimodality nanoprobe, which was composed of Fe_3_O_4_, Au, and anti-EGFR Affibody protein (Au-IONPs) (Yang et al., 2013). In this hetero-nanostuctural nanoprobe, EGFR-targeted IONPs played a major role as T_2_ reporter for MRI. Additionally, Au component was used as positron emission tomography (PET) probe for imaging of EGFR positive tumor cells. The outcomes indicated that Au-IONPs nanoprobe provided significant specificity and high sensitivity for both PET and MRI imaging in the human EGFR-expressing tumor cells (Figure 4B). Moreover, Fe_3_O_4_/Au nanoparticle was conjugated with a single-chain antibody (scFv) to obtain an EGFR-specific MRI bioprobe (scFv@Fe_3_O_4_/Au). The *in vivo* results showed that scFv@Fe_3_O_4_/Au could specifically transfer Fe_3_O_4_/Au to detect EGFR-positive non-small cell lung cancer through MRI method. The scFv@Fe_3_O_4_/Au immunonanoparticles were detected in the cell cytoplasm of EGFR-overexpressing SPC-A1 cells, while very little scFv@Fe_3_O_4_/Au could be detected in EGFR-deficient H69 cells. Therefore, these IONPs-based MRI agents are potential strategies for both selective imaging and cell screening. C225-encapsulated core-shell Fe_3_O_4_@Au were fabricated as a therapeutic nano-system (Fe_3_O_4_@Au-C225) to conduct targeted magneto-photothermal therapy against glioma cells (Lu et al., 2018a). Fe_3_O_4_@Au-C225 integrated magnetic fluid hyperthermia, NIR-induced hyperthermia, and significant specificity was generated by EGFR inhibitor, allowing the glioma specific hyperthermic treatment. Fe_3_O_4_/Ag conjugated with C255 (Fe_3_O_4_/Ag/C225) was fabricated to realize radiation therapy (Zhao et al., 2012). Herein, Fe_3_O_4_ component served as an MRI reporter, while the Ag component assumed the role of a radiotherapy sensitizer. The composite nanoplatform (Fe_3_O_4_/Ag/C225) was used to be an EGFR-targeted tumor tracer for radiation therapy. The *in vitro* experimental results revealed that the enhanced inhibition of human nasopharyngeal carcinoma cell combined with X-ray treatment was found. In a word, this multifunctional nanocomposite Fe_3_O_4_/Ag/C225 might be a potential EGFR-targeted radiosensitizer for treating human nasopharyngeal carcinoma tumor.

Some novel carbon materials are chosen as auxiliaries to construct multifunctional therapeutic agents. Chen et al. took graphene oxide (GO) as a carrier to delivery IONPs and doxorubicin (DOX), and further modified this magnetic graphene oxide with cetuximab (MGO-PEG-CET) (Lu et al., 2018b). This EGFR-targeted magnetic thermo-chemotherapy system could enter high EGFR-expressing CT26 murine colorectal cells by receptor-mediated endocytosis. The *in vivo* and *in vitro* results all demonstrated that EGFR-targeted nanosystems could effectively ablate tumor tissue by synergistic treatment of chemotherapy and photothermal therapy (Figure 4C). In addition, magnetic Fe-filled carbon nanotubes binding with mAb cetuximab could selectively remove EGFR-positive cells from a mixed population of healthy cell lines in about 10 min (Marega et al., 2013). A two-fold increased selective suppression of the EGFR-positive cells was detected compared with EGFR-deficient cells by *in vivo* experiment through an electromagnetic radiation inducing magnetic fluid hyperthermia.

### Metallic Sulfide and Metallic Selenide

In addition, some metallic sulfides and metallic selenides (MxS or MxSe, x = 1–2) have been intensely studied. Herein, some recent reports about zinc sulfide (ZnS), copper sulfide (CuS), silver sulfide (AgS), and cadmium selenide (CdSe) as subjects for EGFR-targeting oncotherapy are summarized in the next section.

**FIGURE 5:**
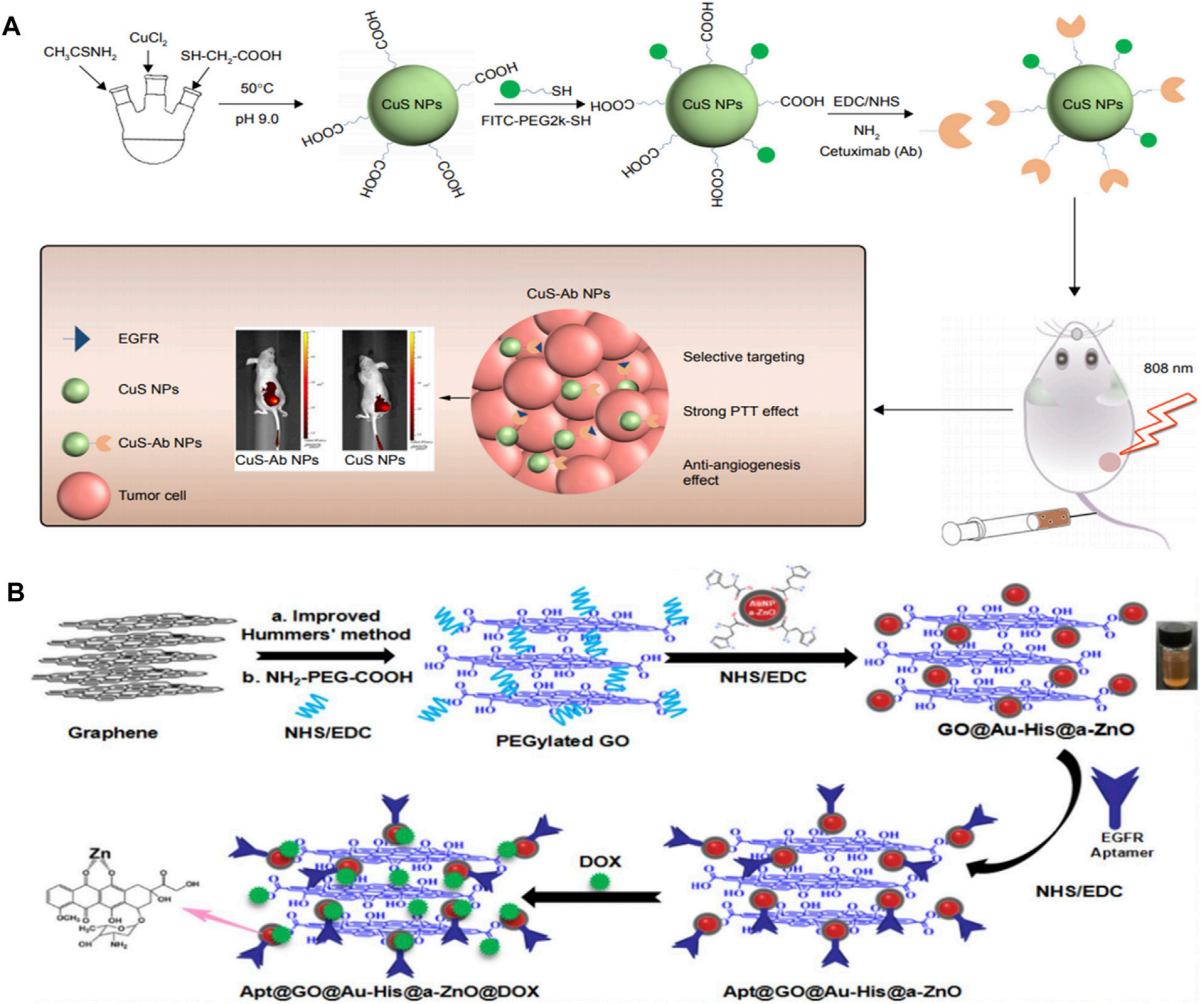
**(A)** Scheme of synthesis and therapy of CuS-Ab NPs. Reproduced from (Deepagan et al., 2012) with permission from Dovepress **(B)** Schematic illustration for the preparation of anti-EGFR aptamer-conjugated and doxorubicin-loaded Apt@GO@Au-His@a-ZnO@DOX NCs. Reproduced from (Chen et al., 2013) with permission from American Chemical Society.

For example, poly (lactic-co-glycolic acid) coated ZnS:Mn^2+^ (PLGA-ZnS) conjugated with cetuximab was used to accomplish targeted imaging and delivery of anti-cancer drugs. As the cell uptake results showed, the uptake of targeted NPs was over 80%, while that of the nontargeted NPs was only 40% (Deepagan et al., 2012). Gong et al. designed a type of QD with core shell structure, in which indium phosphate (InP) and ZnS acted as core and shell, respectively (Wang et al., 2017). The prepared InP/ZnS QDs were endowed with hydrophilia via modifying with amphiphilic block copolymer polylactide-b-poly (ethylene glycol) (PLA-PEG). InP/ZnS@ PLA-PEG micelles were further embellished with an anti-EGFR nanobody (7D12 Nbs) to assess the therapeutic effect of triple-negative breast tumor. As we know, CuS have been demonstrated as potent theranostic nanotool with promising outcomes in the past decade (Yun et al., 2020; Dong et al., 2020). The cetuximab-modified CuS NPs (CuS-Ab NPs) were constructed as a synergistic anti-cancer agent, which could successfully suppress the tumor spread and growth (Li et al., 2018). With the help of cetuximab, CuS NPs were accumulated in tumors rather than in normal tissues. Superior photothermal effect could be obtained under NIR irradiation even at a low power level (0.2 W/cm^2^). Therefore, the designed CuS-Ab NPs fully exploit higher local tumoricidal effect and lower nephric and systemic toxicity by EGFR-targeting with cetuximab (Figure 5A). Additionally, silica coated cadmium selenide quantum dots (CdSe-Silica QDs) conjugated with McAb successfully achieve the specific recognition of EGFR-positive tumor cell lines (Vibin et al., 2017). The resultant QD-Ab finally turned out to be an excellent tumor targeting fluorescent probe, which exhibited much higher internalization efficiency than non-targeted QDs. Over 90% EGFR-targeted QD-Ab probe could enter in the cytoplasm by endocytosis, yet non-targeted QDs showed only 67% internalization. After 4 h of intravenous injection, EGFR-targeted QD-Ab probe specifically accumulated in tumor tissue. Ag_2_S QDs exhibit fluorescence emission maximum within the scope of 650–1,200 nm, making it a promising targeted bioprobe for imaging in neoplastic tissues. In order to solve the lack of specific tumor-targeting capability, anti-EGFR Affibody (ZEGFR:1907)-based Ag_2_S QD (ZEGFR:1907-Ag_2_S QDs) nanoprobes have been successfully synthesized and used for targeted photoacoustic imaging of EGFR-overexpressed tumor (Zhang et al., 2018b). The prepared ZEGFR:1907-Ag_2_S QDs exhibited a sharp and strong absorbance peak at 800 nm along with a weak shoulder absorbance peak in the range of 900–1,100 nm. Two kinds of cell lines with different expression levels of EGFR were chosen to test the targeting specificity cell uptake of ZEGFR:1907-Ag_2_S QDs, including A431 (EGFR-overexpressed) and Bxpc3 with β-actin (EGFR-negative). The uptake of ZEGFR:1907-Ag_2_S QDs was up to 80% in A431 cells due to the prominent targeting and specificity endowed by ZEGFR:1907. In one word, the photoacoustic imaging results showed that ZEGFR:1907-Ag_2_S QDs could detect EGFR-positive tumor cell lines perfectly.

### Metallic Oxideare

In addition, some familiar metallic oxides are also established nanotheranostic platforms combined with EGFR antibody or EGFR KTI against several types of cancer (eg. ZnO NPs) (Muhammad et al., 2011; Chen et al., 2013; Cai et al., 2016; Zhang et al., 2017; Zhou et al., 2017). For example, Wu’s group integrated the histidine-mediate amorphous zinc oxide shells coated Au NPs on graphene oxide to obtain the multifunctional nanocomposites (GO@Au-His@a-ZnO) (Zhang et al., 2019a). The novel nanocomposites were further conjugated with antibody of EGFR aptamer and DOX to accomplish targeting, photothermal, and chemotherapy (Apt@GO@Au-His@a-ZnO@DOX NCs). The maximum loading capacity of DOX was 250 mg/g and the accumulative DOX release rate was 75.4% in PBS pH5.5 under NIR irradiation (808 nm, 1.5 W/cm^2^). Apt@GO@Au-His@a-ZnO@DOX NCs was demonstrated as an excellent photothermal nanomaterial with high photothermal conversion efficiency (*η* = 38%). The resultant Apt@GO@Au-His@a-ZnO@DOX NCs could release antitumor Zn^2+^ ions in the acidic endosome/lysosome of tumor tissue simultaneously. Importantly, Apt@GO@Au-His@a-ZnO@DOX NCs exhibited much higher targeting efficacy and tumor inhibition to EGFR-positive A549 cells than that of GO@Au-His@a-ZnO@DOX NCs according to the laser scanning confocal microscope (LSCM) images for cellular uptake and *in vivo* experiments (Figure 5B).

### Silica Nanoparticles

Except for the above mentioned metallic chalcogenide, silica nanoparticles (SNs) exhibit excellent biodegradability and thus were always chosen as the nanocarriers due to their high porosity and surface area (Wang et al., 2016; Zhang et al., 2019b). Herein, we summarized some recent applications of EGFR-targeted SNs including specific drug delivery, targeted gene therapy, and targeted imaging.

**FIGURE 6:**
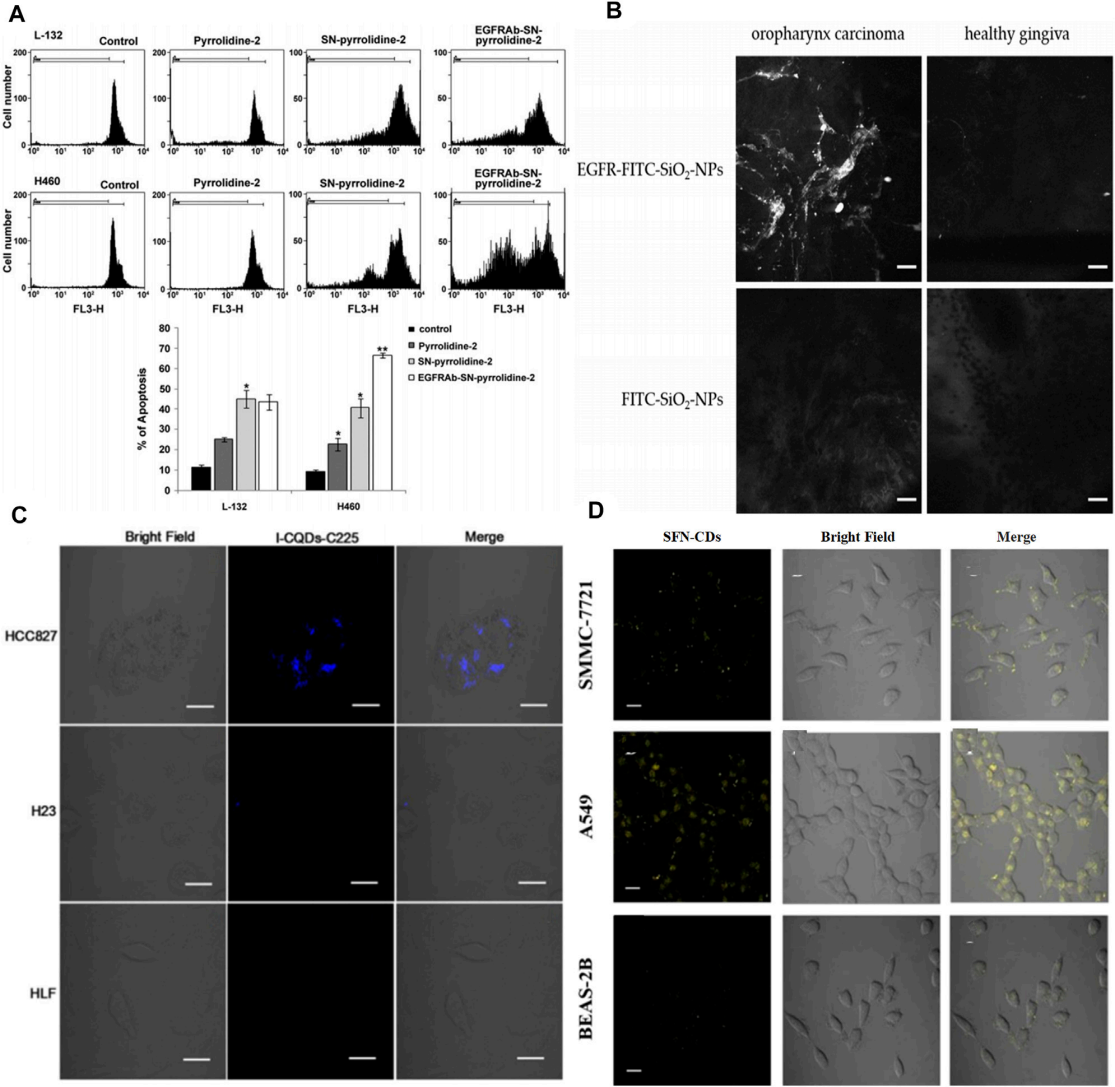
**(A)** Pyrrolidine-2, SN-pyrrolidine-2 and EGFRAb-SN-pyrrolidine-2 induce apoptosis of L-132 and H460 cells. The samples were analyzed by flow cytometry and the fraction of sub-G0/G1 events was detected as a measure of apoptotic cell death. The relative fluorescence intensity measured at the FL3-H channel. Reproduced from (Zhang et al., 2017) with permission from Elsevier **(B)** Confocal laser endoscope imaging of oropharynx carcinoma and healthy gingiva (scale: 25 µm). Reproduced from (Wang et al., 2016b) with permission from Multidisciplinary Digital Publishing Institute **(C)** Laser scanning confocal microscopy images of HCC827 cells (overexpress EGFR), H23 cells (low EGFR), and HLF cells (normal lung cells), incubated with I-CQDs-C225 for 6 h. I-CQDs-C225 was excited with 405-nm laser (scale: 20 μm). Reproduced from (Berlin et al., 2011) with permission from Elsevier **(D)** Fluorescence imaging of SMMC7721, A549 and BEAS-2B cells, incubated with SFN-CDS (0.1 mgmL-1) for 4 h (scale: 20 μm). Reproduced from (Lee et al., 2013) with permission from American Chemical Society.

As for SN-based specific drug delivery, some progress has been made. For instance, polyethylenimine modified mesoporous SNs were conjugated with EGFR antibody (EGFRAb-SN-pyrrolidine-2) to use as a targeted prodrug delivery for effective lung cancer therapy (Sundarraj et al., 2014). According to the flow cytometry results, about 44.57% of EGFRAb-SN-pyrrolidine-2 could be internalized in H460 cells. EGFRAb-SN-pyrrolidine-2 convincingly suppressed H460 cell proliferation compared with EGFR-deficient L-132 cells at the same concentration of 100 μg ml^−1^. And the inhibition rate in EGFRAb-SN-pyrrolidine-2 treated group reached 64% (Figure 6A). Kong et al. synthesized hollow mesoporous silica nanoparticles (HMSNs) and then dressed up HMSNs with amine groups to conjugate with EGF. According to the principal component analysis, the quantity and density of the EGF attachments could be controlled by tuning the EGF concentration at grafting stages (She et al., 2015a; She et al., 2015b). Impressively, they further confirmed that using HMSNs grafted with EGF to deliver 5-FU could overcome acquired 5-fluorouracil (5-FU) resistance. The constructed EGFR-targeted 5-FU nanocarrier (EGF-HMSNs-5-FU) could be specifically internalized in acquired 5-fluorouracil (5-FU) resistance colorectal cell line (SW480/DAR) abundantly through a receptor-mediated endocytosis, thus resulting in cell death through S phase arrest (Chen et al., 2015).

More interestingly, EGFR-targeted MNs also could deliver small interfering RNA (siRNA) to directly silence the gene expression with complementary messenger RNA (mRNA) sequence, particularly the reversal of drug resistance (Chen et al., 2018; Cao et al., 2020; Tieu et al., 2021). The inhibition ratio was up to 74 ± 6% when incubated with EGFR-pSiNPs through the dual treatments of chemotherapy and genetic therapy. EGFR-pSiNPs were further used as an efficient vehicle to deliver camptothecin to solve its poor water-soluble and toxic side effects (Landgraf et al., 2020). Shao et al. developed a targeted gene-drug co-delivery system based on polyamidoamine (PAMAM) mediated HMSNs (Zhang et al., 2020a). CRISPR/Cas9 was chosen to provide gene therapy, meanwhile, Sorafenib acts as molecular target by specifically inhibiting EGFR or VEGFR2 on hepatocellular carcinoma. Importantly, the co-delivery therapeutic systems caused 85% specific targeting tumor inhibition *in vivo* models. Furthermore, the nanocomplex showed high accumulation at the tumor site *in vivo* and exhibited good safety with no damage to major organs. Ji et al. also proved that using MSNs to deliver cetuximab and DOX could accomplish effective management of EGFR-mutant lung cancer and overcome acquired drug resistance (PC9-DR). The resultant MSNs have the specific surface area of 887.9 m^2^/g and the pore size of 2.5 nm, which is suitable to carry cetuximab, gefitinib, and DOX into cells with high EGFR expression by endocytosis. Significant effect upon inhibiting PC9-DR xenograft tumor growth was observed; the weight of the xenograft tumors decreased to 0.1 g, indicating the potential ability to solve drug resistance (Wang et al., 2016). Brinker et al. recently synthesized the mesoporous silica nanoparticles-supported lipid bilayers conjugating of gemcitabine to endow active EGFR targeting (Durfee et al., 2016). They emphasized on the factors influencing size uniformity and long-term stability in complex biological media. As expected, compared with EGFR-negative control cells, significant bonding specificity was observed and the maximal binding was realized at 30 min of incubation by flow cytometry combined with fluorescence microscopy. Additionally, Brennan et al. proved the ultrasmall SNs (<8 nm) as drug delivery to improve the biological characteristics and therapeutic properties of gefitinib. The ultrasmall size of SNs was conducive to increase tumor accumulation and dominant renal excretion (Weissleder et al., 2014; Bregoli et al., 2016).

EGFR-labeled MSNs were also used as directional carriers of nano-contrast agents for real-time tumor detection. Gadolinium (Gd^3+^) is well known as an exogenous contrast agent with strong T1-weighted image signal intensity, however, high doses and indiscriminate accumulation result in terrible side-effects for normal tissues. Therefore, EGFR antibody conjugated SNs were utilized for geometric confinement of small molecule Gd-chelate (Sinha et al., 2017). The longitudinal MR relaxivity (r1) value of before and after EGFR antibody conjugation on Gd-chelate grafted MSNs were calculated as 22.19 and 19.39 mM^−1^S^−1^, respectively. Besides, the fluorophore fluorescein (FITC) is an important auxiliary to implement contrast enhancement. FITC-labeled SNs with the attachment of anti-EGF-receptor-antibodies (Alexa Fluor^®^555) were built as a novel contrast agent (AF555-EGFR-FITC-SiO_2_-NPs) (Watermann et al., 2019). It could real-time define tumor borders, contributing to easier surgical intervention in the treatment of carcinoma of the head and neck (Figure 6B). Moreover, Nonell et al. provided a strategy of combining EGFR-targeted MSNs and zinc phthalocyanine (ZnPcOBP) for selective photodynamic therapy (Er et al., 2018). The C225-labled MSNs were applied as a photosensitizer carrier. The quantum yield of singlet oxygen production of undecorated ZnPcOBP was about 0.60 in toluene. Comfortingly, the fatality rate of ZnPcOBP-loaded-MSNs coated with PEG and C225 (ZnPcOBP-loaded-MSNP5) on MIA PaCa-2 cells reached 80%, indicating that abundant photosensitizer molecules were delivering into intracellular membranes via EGFR-targeted cell uptake. This strategy based on EGFR-targeted MSNs could avoid unwanted photo-induced damage to normal tissue cells.

### Carbon Dots

The fabrication and bioimaging applications of carbon dots (CQDs) have rapidly advanced in recent years because of their photostablity and chemical stability. Importantly, CQDs contain no heavy metals, suggesting lower biotoxicity than semiconductor QDs (Bhirde et al., 2009; Berlin et al., 2011; Sano et al., 2012; Lee et al., 2013; Rungnim et al., 2016; Sengupta et al., 2015). When CQDs were modified with EGF antibody, the functionalized CQDs have been invested with specific targeting. To improve spatial resolution and enhance tissue penetration of fluorescence imaging, the incorporations of CT or MRI agents into EGFR-targeted CQDs could obtain the multimode imaging nanoplatforms. In the clinic, iodine and Gd-chelates are traditional CT and MRI contrast agents that are always chosen to combine with novel nanomaterials. For instance, Zhu et al. prepared the cetuximab-conjugated iodine doped CQDs ((I-CQDs-C225) by a “one-pot” hydrothermal method to construct dual fluorescent/CT bioprobe for targeted imaging (Su et al., 2018). I-CQDs-C225 showed bright blue fluorescence with the quantum yield of 18%, which was similar to that of I-CQDs without antibodies. The CT image brightness of I-CQDs-C225 was nearly equal to or brighter than those iodixanol (commercial contrast agents) at the same concentration. I-CQDs-C225 were abundantly internalized into the lysosomes of HCC827 cells (EGFR-overexpression non-small lung cells) and showed a strong blue fluorescence after incubation for 6 h (Figure 6C). Gd(III)-encapsulated CQDs were conjugated with Ac-Cys-ZEGFR:1907 (a kind of EGFR antibodies) forming an effective MRI contrast agent (Wu et al., 2020). This ideal bioprobe maintained the optimal T1 relaxivity without obvious cellular toxicity. Dong’s group constructed sulforaphane-functionalized carbon dots (SFN-CDs) for EGFR-overexpressing cancer cell targeted imaging and inhibition (Lu et al., 2019). SFN-CDs showed a strong yellow fluorescence at 547 nm, which could effectively avoid the influence of autologous fluorescence in organisms. The results of *in vitro* experiments, targeted imaging, and effective apoptosis of cancer cells were caused by the specific internalization of SFN-CDs (Figure 6D). Interestingly, Zhang et al. recently prepared red-emitting CDs-embedded epitope imprinted polymer (C-MIP) for fluorescence imaging and EGFR-positive tumor cell identification (Zhang et al., 2020b). The fluorescence of C-MIP was quenched with the determination limit of 0.73 μg ml^−1^, when C-MIP specifically bonded to the epitopes of EGFR through their imprinted cavities. Erlotinib mediated nitrogen doped CDs (NCDs) also proved that this nanosystem possessed an effective capability for targeted fluorescence imaging of pancreatic cancer cells (Devi et al., 2020). It is worth noting that nitrogen doped content had an effect on photoluminescence of NCDs, and thus NCDs (citric acid: urea = 1:1 by mass) exhibited the strongest fluorescence intensity. EGFR-NCDs by coating bovine serum albumin (BSA) retained their fluorescence even with increased fluorescence intensity to a small extent.

## Future Perspectives and Conclusion

The rapid advance of nanotechnology in recent years has provided progressive strategies for cancer therapy. Various advantages of novel iNPs have been identified as multifunctional nanotherapeutics, especially molecular targeted therapy for cancers. Metal nanoparticles and nonmetallic nanoparticles have unique properties, such as easy accumulation in tumor cells, fluorescence imaging, enhanced Raman scattering, photothermal, and antimicrobial properties. A large number of biomedical applications have been successfully demonstrated *in vitro*. But there is still room for further improvement so that the nanoparticles can be suitable for clinical trials and applications. Some of the challenges that still need to be addressed include improving the stability of nanoparticles in a variety of complex *in vivo* environments, designing proper functionalization to improve biocompatibility and reducing cytotoxicity, effective cost control, and optimization of synthesis protocols. This paper sums up the description and the benefits of different EGFR-loaded nanotechnological platforms based on iNPs.

As we previously described, EGFR-targeted iNPs have a solid role in imaging diagnosis and cancer therapy. However, these EGFR-targeted antineoplastic agents still have a great distance from nanomedicine in lab to clinical reality and there are many unresolved issues to be elucidated. First of all, regarding the synthesis, the controllable preparation of iNPs with uniform size, high crystallinity, and good morphology is important. Second, combining EGFR-mediated iNPs with the guidance of fluorescence, CT, or MRI imaging has a great perspective to achieve more satisfying therapeutic effect. Therefore, the construction of imaging-guided and EGFR-targeted therapy in one nanoplatform still needs to be further explored to achieve drug delivery monitoring, tumor localization, and treatment effect monitoring. Additionally, whether and why the combination of iNPs with EGFR-KIT or McAb could overcome the drug resistance to a certain extent and suppress the progression of point mutation in the DNA sequence is an urgent issue to understand. Moreover, the pharmacodynamic and pharmacokinetic properties of inorganic nanoparticles are immature. As a consequence, numerous comprehensive studies need to be carried out to scrutinize the pharmacokinetic, biodistribution, and safety profiles of iNPs. Given the interactions between iNPs and immune system in body, the immunotoxicity of iNPs should be further assessed in future.

In a word, great efforts are required to meet the challenges in implementing the transformation from nanomedecine to clinical trials. With profound exploration, iNPs will revolutionize molecular targeted cancer therapy in the near future.
